# The Experience of a Multidisciplinary Clinic in the Management of Early-Stage Breast Cancer Patients Receiving Trastuzumab Therapy: An Observational Study

**DOI:** 10.1155/2012/135819

**Published:** 2012-09-25

**Authors:** Susan Dent, Sean Hopkins, Nadine Graham, Christopher Johnson, Angeline Law, Michelle Campbell, Freya Crawley, Kathleen Allen, Michele Turek

**Affiliations:** ^1^Division of Medical Oncology, Department of Medicine, University of Ottawa, The Ottawa Hospital Cancer Centre, Ottawa, ON, Canada K1H 8L6; ^2^Department of Pharmacy, The Ottawa Hospital Cancer Centre, Ottawa, ON, Canada K1H 8L6; ^3^The Ottawa Hospital Cancer Centre, Ottawa, ON, Canada K1H 8L6; ^4^Division of Cardiology, Department of Medicine, University of Ottawa, The Ottawa Hospital, Ottawa, ON, Canada K1H 8L6

## Abstract

*Background*. We established a dedicated cardiac oncology clinic in 2008 for the rapid diagnosis and treatment of cardiotoxicity related to cancer therapy. In this retrospective observational study, we report on clinical outcomes in women with early-stage breast cancer (EBC) referred to this clinic. *Methods*. Patients with EBC treated with chemotherapy/trastuzumab and referred between October 2008 and December 2010. Data included patient demographics, staging, cancer treatment/completion, dose delays, left ventricular ejection fraction (LVEF) and cardiac treatment. *Results*. Forty eight patients: median age 55.5 years, stage I/II disease (77%) and HER-2 positive (98%). The majority of women (*n* = 32) were referred for decreases in LVEF (from baseline). Overall, 37 (77%) patients experienced at least one drop in LVEF while on treatment, of which 22 patients (59%) experienced a ≥10 percentage point drop. The majority of patients (30/37; 81%) experienced declines in LVEF while on trastuzumab. Interventions included trastuzumab delays (*n* = 16/48; 33%) and cardiac medication (12/48: 25%). A total of 81% of patients completed ≥90% of trastuzumab therapy and 15% of patients discontinued therapy due to cardiotoxicity. *Conclusion*. The majority of patients referred to our clinic completed therapy. Further studies are needed to determine the impact of this multidisciplinary approach on treatment completion and cardiac outcomes.

## 1. Introduction

Breast cancer is the most common malignancy for women in North America [1]. The use of anthracycline-based regimens in the adjuvant setting has been shown to improve overall survival [2], but with an increased risk of cardiotoxicity. Approximately 20% [3] of women with breast cancer overexpress the HER-2 protein, a member of the epidermal growth factor receptor family. Trastuzumab, a monoclonal antibody that targets the HER-2 protein, was initially tested in women with metastatic breast cancer in combination with anthracycline-based chemotherapy. Concurrent administration of anthracycline/trastuzumab led to significant response rates but high rates of congestive heart failure (28%) [4]. 

In four large randomized control trials (NSABP B31, NCCTG 9831, HERA, BCIRG 006) [5–7] of early stage breast cancer, significant improvements in both disease free (HR = 0.60) and overall survival (HR = 0.66) were observed in patients who received chemotherapy and trastuzumab (following anthracyclines) compared with those who received chemotherapy alone [8, 9]. This led to the adoption of one year of trastuzumab (every 3 weeks for 18 cycles) as the standard of care for women with early stage HER-2-positive breast cancer. While there was significantly less cardiotoxicity observed with trastuzumab in the adjuvant breast trials (congestive heart failure 2.5%) [10], clinicians have perceived higher rates in clinical practice. In a retrospective single-institution study of 105 women with HER-2-positive early stage breast cancer, we reported dose delays of trastuzumab in 30% (*n* = 32) of patients [11]. In this study, 13% of patients experienced ≥10 percentage point declines in left ventricular ejection fraction (LVEF) to less than 55%, although with appropriate medical management, the majority of patients (89%) were able to complete their targeted treatment. Other case series have reported higher discontinuation rates (20%) [12] for trastuzumab. Early discontinuation of trastuzumab could potentially compromise the clinical benefit of this treatment. Outside the clinical trial setting, there has been no standardized approach to the treatment of women who have sustained cardiac dysfunction while on trastuzumab therapy. In addition, there is little guidance in the literature on who should manage patients that experience cardiotoxicity as a result of their breast cancer treatments.

In March of 2007, a panel of experts from across Canada convened in Toronto to develop a Canadian consensus guideline to assist clinicians in the identification and treatment of women experiencing cardiac dysfunction while on trastuzumab [13]. The panel recommended that women with early stage breast cancer exposed to trastuzumab should be managed in close collaboration with cardiologists. It was clear from this meeting that many medical oncologists did not have the clinical expertise or knowledge necessary to manage the cardiotoxicities experienced by these women. It was also apparent that cardiologists needed a better understanding of the clinical benefit of targeted therapies when making decisions with regards to cardiac management.

In 2008, we established a multidisciplinary cardiac oncology clinic to facilitate the rapid diagnosis and treatment of cardiac complications secondary to cancer therapy. The multidisciplinary team consisted of one medical oncologist, three cardiologists, one pharmacist, and one nurse. While the initial focus of this clinic pertained to women with early stage breast cancer exposed to chemotherapy +/− trastuzumab, the widespread adoption of targeted therapies in oncology has subsequently led to the referral of a much broader patient population. Between October 1, 2008 and December 31, 2010, 188 patients were referred to this clinic, the majority (*n* = 127; 68%) of whom were breast cancer patients (early and advanced stages). 

The purpose of this retrospective observational study is to report on the clinical and cardiac outcomes in women with early stage breast cancer receiving trastuzumab, who were referred to our multidisciplinary cardiac oncology clinic. This study was approved by the Ottawa Hospital Research Ethics Board.

## 2. Patients and Methods

Early stage breast cancer patients receiving trastuzumab who were referred to the cardiac oncology clinic between October 1, 2008 and December 31, 2010 were included in this study. Data collection included patient demographics, staging, cancer treatment and completion rates, LVEF, and cardiac treatment. Data with regards to dose delays and early termination of chemotherapy or trastuzumab were obtained. Patients were referred to the cardiac oncology clinic by their primary oncologist if they had a LVEF <50% presystemic therapy, a decline in LVEF to <50% during treatment, a decline in LVEF by ≥10 percentage points during treatment, concerns regarding cardiotoxicity, or evidence of symptomatic congestive heart failure. Changes in LVEF were calculated based on percentage point differences from baseline assessment. MUGA scans and echocardiograms (to assess LVEF) were done prior to commencing chemotherapy and every 3 months for patients on trastuzumab therapy as per institution policy. Additional cardiac investigations were performed at the discretion of the treating physician.

## 3. Results

 Between October 2008 and December 2010, 48 women with early stage breast cancer receiving potentially cardiotoxic cancer therapy (anthracyclines/trastuzumab) were referred to the cardiac oncology clinic. Baseline patient characteristics and demographics are shown in Table 1. The median age of patients was 55.5 years and the majority of patients had stage I/II disease (77%). Most patients had estrogen-receptor-positive (67%) and HER-2 positive disease (98%). Patients had a median of at least one (*r*: 0–7) cardiovascular risk factor, 27% having a BMI ≥30 and 23% having a smoking history. 

All patients received systemic therapy as outlined in Table 2. Approximately 85% of women were exposed to anthracyclines and trastuzumab, while 15% received nonanthracycline chemotherapy and trastuzumab. The median number of trastuzumab cycles delivered was 18 (*r*: 3–18) and the median number of anthracycline cycles was 4 (*r*: 1–6). Reasons for referral to the cardiac oncology clinic included decline in LVEF (*n* = 32; 66.7%), pretreatment assessment (3), cardiomyopathy (3), valve disease (2), congestive heart failure (1), tachycardia (1), hypokinesis (1), dyspnea (1), palpations (1), LV dysfunction (1), LV enlargement (1), and abnormal echocardiogram (1).

Cardiac imaging was performed as outlined in Table 3. The majority of patients had cardiac imaging (MUGA or echocardiogram) at baseline (*n* = 45; 94%) with median LVEF measurements of 63% (*r*: 50–80%). Overall, 37 (77%) patients experienced at least one drop in LVEF, of which 22 (59%) patients experienced a ≥10 percentage point drop from baseline. The majority of these patients (30/37; 81%) experienced a decline in LVEF during trastuzumab, while 5 (13%) patients experienced a decline in LVEF following completion of all systemic therapy. 

Treatment outcomes are outlined in Table 4. A total of 94% of patients successfully completed their recommended chemotherapy. The majority of patients (*n* = 39/48; 81%) successfully completed ≥90% (16–18 cycles) of their recommended trastuzumab therapy. One third of patients (*n* = 16; 33%) experienced delays in trastuzumab therapy due to declines in LVEF (median 3 weeks; *r*: 3–12 weeks), of which 12/16 (75%) patients successfully completed their targeted therapy (5 with cardiac medication). Overall, 12 (25%) patients received cardiac medication: 10 ACE inhibitor, 6 beta blocker, and 2 other cardiac drugs. Median cardiac oncology clinic visits were 3 (*r*: 1–8).

Approximately, one third of patients (*n* = 18; 37%) had at least one LVEF measurement of less than 50% (median 45.5%; *r*: 19–49%) as outlined in Table 5. The majority of declines in LVEF occurred during trastuzumab therapy (*n* = 15/18, 83%), of which 10/15 (67%) had recovery of their LVEF to >50% (5 with cardiac medication). Eight of 18 (44%) patients successfully completed ≥90% (16–18 cycles) of trastuzumab. Cardiac intervention for these patients included dose delays (*n* = 5; 28%) and cardiac medication (*n* = 8; 44%). 

A total of 7 (15%) patients with a median LVEF of 31% (*r*: 19–45%) discontinued trastuzumab permanently due to cardiotoxicity. These patients had normal baseline cardiac function (median LVEF 57%; *r*: 50–65%) and all 7 patients had previous exposure to anthracyclines (median dose: 408 mg; *r*: 240–684 mg). These patients had a median of one (*r*: 0–2) cardiac risk factor. Three (43%) of 7 patients were administered cardiac medication and 3 (43%) patients had an LVEF recovery to above 50% (one with cardiac medication). As of December 2010, one patient had a fatal myocardial infarction 16 months after stopping trastuzumab (5 cycles) due to cardiotoxicity.

## 4. Discussion

The evolution of personalized medicine has led to an increasing interest in the development and testing of targeted therapies in oncology. While cancer professionals have been well versed in the identification and treatment of toxicities associated with chemotherapy, there is less understanding of the short- and long-term consequences associated with targeted agents. The widespread adoption of trastuzumab in combination with chemotherapy has led to significant improvements in the clinical outcomes of women with both early- and advanced-stage breast cancer. While congestive heart failure rates (2.5%) [10] in the adjuvant trastuzumab breast trials have been modest, clinicians have observed more subtle signs of cardiotoxicity (e.g., decreases in LVEF) in the nonclinical trial setting, potentially leading to early discontinuation of treatment. 

In this retrospective observational study, we report on the clinical outcomes of early stage breast cancer patients treated with chemotherapy and trastuzumab who were referred to a dedicated cardiac oncology clinic. While the majority of patients were referred to the clinic for a decline in LVEF during trastuzumab treatment, many of these women had previous exposure to anthracyclines, thus increasing their risk of experiencing cardiotoxicity. Completion rates for chemotherapy were high for early stage breast cancer patients seen in the cardiac oncology clinic (94%). Although several patients experienced substantial declines in LVEF (≥20 percentage points), 81% completed 16 or more cycles of trastuzumab and only 15% (*n* = 7) discontinued therapy permanently due to cardiotoxicity. Approximately, 35% of patients experienced short delays in trastuzumab therapy due to cardiotoxicity; however, with appropriate medical management and surveillance, 77% completed treatment. 

In summary, the majority of women with early stage breast cancer who were referred to the cardiac oncology clinic completed therapy. The discontinuation rate observed in our study (15%) is higher than reported with the HERA trial (4.3%) [9], but similar to that reported in the NCCTG 9831 study (18.9%) [14]. The lower rates of discontinuation in the HERA trial likely reflect the higher baseline cardiac function (LVEF ≥55%) required for study entry compared to the NCCTG trial (LVEF ≥50%). In our study, only one of seven patients who discontinued trastuzumab prematurely had a baseline LVEF <55% and thus, one can speculate that other risk factors for cardiotoxicity were present that were not detected with baseline medical history and cardiac imaging. Better predictors of cardiotoxicity such as cardiac biomarkers and novel imaging techniques are needed for early detection of cardiotoxicity.

In 2010, we established the Canadian Cardiac Oncology Network with the mission of optimizing cardiac care for cancer patients receiving potentially cardiotoxic therapies. Through this multidisciplinary approach, we hope to gain a better understanding of cardiac complications of cancer treatments and develop early detection and intervention strategies to optimize cardiac health in these patients.

## Figures and Tables

**Table 1:** Patient demographics.

	Early stage breast cancer patients
	*N* = 48
Median age at diagnosis	55.5 years (*r*: 33.5–76.6 years)
Disease stage	
(i) I	14 (29.2%)
(ii) II	23 (47.9%)
(iii) III	11 (22.9%)
Receptor status	
(i) ER positive	32 (66.7%)
(ii) PR positive	25 (52.1%)
(iii) HER/2 positive	47 (97.9%)
(iv) HER/2 borderline	1 (2.1%)
Node status	
(i) Node positive	16 (33.3%)
Cardiac risk factors (median)	1 (*r*: 0–7)
(i) Obese (BMI > 30)	13 (27.1%)
(ii) Smoker	11 (22.9%)
(iii) Hypertension	10 (20.8%)
(iv) Hypercholesterolemia	2 (4.2%)
(v) Coronary artery disease	1 (2.1%)
(vi) Diabetes	2 (4.2%)

**Table 2:** Treatment regimens.

	Early stage breast cancer patients
	*N* = 48
	*N* (%)
(i) Anthracycline CT + T	41 (85.4%)
(ii) Non-anthracycline CT + T	7 (14.6%)
Median anthracycline CT dose	461 mg (*r*: 119–1424 mg)
Median CT cycles	6 (*r*: 2–8)
Median anthracycline CT cycles	4 (*r*: 1–6)
Median length of T treatment	18 cycles (*r*: 3–18)

CT: chemotherapy.

T: trastuzumab.

**Table 3:** Cardiac measures.

	Early stage breast cancer patients
	*N* = 48
Prechemo-LVEF testing	
(i) ECHO	24 (50%)
(ii) MUGA	21 (43.8%)
(iii) None	3 (6.2%)
Median prechemo-LVEF	63% (*r*: 50—80)
LVEF evaluation (from baseline)	
(i) Decreased LVEF	37 (77.1%)
(ii) Stable LVEF	7 (14.6%)
(iii) Not evaluable	4 (8.3%)
(a) No baseline LVEF	3 (6.2%)
(b) No followup LVEF	1 (2.1%)
Maximum percentage point (%) decrease in LVEF from baseline	*N* = 37
(i) <5	8 (21.6%)
(ii) 5–9.9	7 (18.9%)
(iii) 10–19.9	14 (37.8%)
(iv) ≥20	8 (21.6%)
Median percentage point decrease in LVEF (%)	11.9 (*r*: 1.8–33)
Absolute decline in LVEF <50%	18 (37.5%)
Timing of decreased LVEF	*N* = 37
(i) During CT	1 (2.7%)
(ii) During CT and T	1 (2.7%)
(iii) After CT	1 (2.7%)
(iv) After CT, on T	29 (78.4%)
(v) After CT and T	5 (13.5%)

**Table 4:** Treatment outcomes.

	Early stage breast cancer patients
	*N* = 48
Chemotherapy	
Chemotherapy completion	
(i) Complete	45 (93.7%)
(ii) Discontinued (non cardiotoxicity)	1 (2.1%)
(iii) Discontinued (Patient choice)	2 (4.2%)
Chemotherapy cycle delays	
(i) Cardiotoxicity	1 (2.1%)
(ii) Completion post delays	1 (100%)
Trastuzumab	
Trastuzumab completion	
(i) Complete	37 (77.1%)
(ii) Discontinued (cardiotoxicity)	7 (14.6%)
(iii) Discontinued (non cardiotoxicity)	2 (4.2%)
(iv) Discontinued (Patient choice)	2 (4.2%)
≥90% trastuzumab completion (16–18 cycles)	39 (81.3%)
Trastuzumab cycle delays	
(i) Cardiotoxicity	16 (33.3%)
(ii) Completion post delays	12 (75%)
Cardiac medication	12 (25%)
(i) ACE inhibitor	10 (83.3%)
(ii) Beta blocker	6 (50%)
(iii) Other cardiac drugs	2 (16.7%)

**Table 5:** Patients with at least one absolute LVEF < 50%.

	(*N* = 18)
Patient demographics	
Disease Stage	
(i) I	6 (33.3%)
(ii) II	7 (38.9%)
(iii) III	5 (27.8%)
Cardiac Risk Factors (median)	1 (*r*: 0–3)
(i) Obese (BMI > 30)	5 (27.8%)
(ii) Smoker	4 (22.2%)
(iii) Hypertension	3 (16.7%)
(iv) Diabetes	1 (5.6%)
Treatment regimen	
(i) Anthracycline CT + T	17 (94.4%)
(ii) Nonanthracycline CT + T	1 (5.6%)
≥90% trastuzumab completion (16–18 cycles)	8 (44.4%)
Cardiac measures and intervention	
Prechemo-LVEF testing	
(i) ECHO	6 (33.3%)
(ii) MUGA	10 (55.6%)
(iii) None	2 (11.1%)
Median prechemo-LVEF	61.5 (*r*: 50–72)
Percentage point (%) decrease in LVEF from baseline to <50%	
(i) <5	2 (11.1%)
(ii) 5–9.9	1 (5.6%)
(iii) 10–19.9	2 (11.1%)
(iv) ≥20	8 (44.4%)
(v) Unknown (no baseline)	2 (11.1%)
(vi) ≥10 percentage points decrease to LVEF <50%	10 (55.6%)
(vii) Median LVEF <50%	45.5% (*r*: 19–49%)
Timing of absolute LVEF <50%:	
(i) During CT and T	2 (11.1%)
(ii) After CT	1 (5.6%)
(iii) After CT, during T	13 (72.2%)
(iv) After CT and T	2 (11.1%)
LVEF outcome	
(i) Recovered LVEF to ≥50%	13 (72.2%)
Cardiac medication	*N* = 8
(i) ACE inhibitors	5 (62.5%)
(ii) Beta blockers	4 (50%)
(iii) Other cardiac medication	2 (25%)
Dose delays	
(i) *T* delay	5 (27.8%)

CT: chemotherapy.

T: trastuzumab.
